# Application of *Trichoderma harzianum*, 6-Pentyl-α-pyrone and Plant Biopolymer Formulations Modulate Plant Metabolism and Fruit Quality of Plum Tomatoes

**DOI:** 10.3390/plants9060771

**Published:** 2020-06-19

**Authors:** Petronia Carillo, Sheridan L. Woo, Ernesto Comite, Christophe El-Nakhel, Youssef Rouphael, Giovanna Marta Fusco, Assunta Borzacchiello, Stefania Lanzuise, Francesco Vinale

**Affiliations:** 1 Department of Environmental, Biological and Pharmaceutical Sciences and Technologies, University of Campania “Luigi Vanvitelli”, Via Vivaldi 43, 81100 Caserta, Italy; petronia.carillo@unicampania.it (P.C.); giovannamarta.fusco@unicampania.it (G.M.F.); 2 Department of Pharmacy, University of Naples Federico II, 80131 Naples, Italy; 3 Task Force on Microbiome Studies, University of Naples Federico II, 80055 Portici, Italy; 4 National Research Council, Institute for Sustainable Plant Protection, 80055 Portici, Italy; frvinale@unina.it; 5 Department of Agricultural Sciences, University of Naples Federico II, 80055 Portici, Italy; ernesto.comite@unina.it (E.C.); Nakhel_Christophe@hotmail.com (C.E.-N.); youssef.rouphael@unina.it (Y.R.); stefania.lanzuise@unina.it (S.L.); 6 National Research Council, Institute for Composite Polymers and Biomaterials, 80125 Napoli, Italy; assunta.borzacchiello@cnr.it; 7 Department of Veterinary Medicine and Animal Productions, University of Naples Federico II, 80137 Naples, Italy

**Keywords:** microbial biostimulant, non-microbial biostimulant, carboxymethyl cellulose, Pluronic F-127, amino acids, lycopene, GABA, *Solanum lycopersicum* L.

## Abstract

Many *Trichoderma* are successfully used to improve agriculture productivity due to their capacity for biocontrol and to stimulate plant growth and tolerance to abiotic stress. This research elucidates the effect of applications with *Trichoderma harzianum* strain T22 (T22), or biopolymer (BP) alone or in combination (BP + T22 or BP + 6-pentyl-α-pyrone (6PP); a *Trichoderma* secondary metabolite) on the crop performance, nutritional and functional quality of greenhouse tomato (*Solanum lycopersicum* L. cultivar Pixel). T22 elicited significant increases in total yield (+40.1%) compared to untreated tomato. The content of lycopene, an important antioxidant compound in tomatoes, significantly increased upon treatment with T22 (+ 49%), BP + T22 (+ 40%) and BP + 6PP (+ 52%) compared to the control. T22 treatments significantly increased the content of asparagine (+37%), GABA (+87%) and MEA (+102%) over the control; whereas BP alone strongly increased GABA (+105%) and MEA (+85%). The synthesis of these compounds implies that tomato plants are able to reuse the photorespiratory amino acids and ammonium for producing useful metabolites and reduce the pressure of photorespiration on plant metabolism, thus optimizing photosynthesis and growth. Finally, these metabolites exert many beneficial effects for human health, thus enhancing the premium quality of plum tomatoes.

## 1. Introduction

Until a few decades ago, agriculture intensification was the principal strategy proposed to ensure food security and socio-economic development, guaranteeing continuous productivity from season to season, in optimal and sub-optimal conditions [1]. However, at present, it is clear that to feed the growing global population, expected to reach 10 billion people in 2050, it is necessary not only to reconcile an increased need for agricultural productivity, but to also resolve the extensive use of chemical products (i.e., fertilizers and pesticides) and their negative impact on the environment and human health [1,2]. According to the FAO [3], this objective can only be achieved by being attentive to nutritional and consumption choices and by employing sustainable agricultural management practices. A promising and eco-friendly strategy for agriculture could be the integrated use of diverse non-chemical methods and products in cropping systems, which includes the implementation of plant biostimulants (PBs), based on beneficial microorganisms and molecules of natural origin [4]. PBs include various bioactive substances, formulations of compounds or microorganisms, such as humic and fulvic acids, macro and microalgal extracts, protein hydrolysates, chitosans and silicon, as well as mycorrhizal fungi and plant growth-promoting microorganisms able to improve plant growth, vigor and yield even under suboptimal conditions [5,6]. Other plant beneficial microorganisms, in particular *Trichoderma* spp., are able to control pathogens and pests [7,8], as well as to increase plant nutrient use efficiency (NUE) [9], synchronize the up-regulation of photosynthetic capacity and carbohydrate metabolism [10,11], plus improve plant growth, yield and produce quality [12,13,14]. In particular, *Trichoderma asperellum*, *Trichoderma atroviride*, *Trichoderma harzianum*, *Trichoderma virens* and *Trichoderma viride* have been demonstrated to act as plant biostimulants, attributed to their capacity to stimulate NUE, and consequently increase plant vigor and tolerance to abiotic stresses [9,12,13,15,16]. It is important to specify that NUE does not only depend upon nutrient uptake by the plant, in particular of nitrogen, but it is also affected by the nutrient assimilation of organic compounds, as well as their translocation throughout the plant [17,18]. The beneficial activity of fungi belonging to *Trichoderma* depends upon their ability to establish a molecular dialogue with the plant, by actively secreting and exchanging small molecules (i.e., hormone-like compounds), able to re-modulate gene expression and metabolic processes in plant roots and shoots [19,20]. Some bioactive metabolites can directly increase elemental (i.e., Ca, Fe, K, Mg, Mn, P and Zn) uptake in the roots [21,22,23]. However, these metabolites are also responsible for the direct stimulation of root growth and development, thus enhancing the surface area and root extension in the soil environment, which improves NUE [11,24,25], by using mechanisms similar to those of other beneficial microbes in the root community (i.e., arbuscular mycorrhiza and plant growth-promoting rhizobacteria) [26,27,28].

*Trichoderma harzianum* T-22 is able to colonize the roots of most plant species independently of the soil type [14,29]. Many *Trichoderma* are good producers of a variety of secondary metabolites, some which demonstrate antibiotic proprieties and plant growth effects. A well-studied volatile compound, 6-pentyl-α-pyrone (6PP), responsible for the coconut-like odor of *Trichoderma* cultures [30], has demonstrated moderate antibiotic activity in vitro and in vivo against several phytopathogens [7,31,32], but interestingly, when used in combination with fungus cell wall degrading enzymes, synergistic effects were noted in the inhibition of the target fungal pathogens by 60% [7,10]. In addition, when 6PP was applied exogenously at very low concentrations (e.g., by 1 ppm root applications or 1 µM shoot spray), to wheat or tomato seedlings and etiolated pea stems, as well as to substrates with canola or tomato seeds, an auxin-like effect was observed in plant growth and development [33].

The possibility to obtain new biostimulant preparations that optimize the application of beneficial microorganisms, or the controlled release of their bioactive metabolites, could represent an innovative approach for eco-sustainable agriculture. To this end, the use of natural and/or synthetic biopolymer materials could be considered as carriers for *Trichoderma* and plant growth-promoting products to facilitate the formulation, delivery and activity of these active compounds [34,35], plus overcome the instability of the *Trichoderma* spore suspensions or the metabolite in water solutions. For the design of biopolymer formulations, it is very common to use carbohydrate polymers that present advantages, such as biocompatibility and biodegradability, and possess a high capacity to absorb water plus the possibility to load up a great variety of nutrients and agricultural compounds in their structure [36]. Among the polysaccharides, carboxymethyl cellulose (CMC), a polyanion derivative of cellulose commonly used as thickener and emulsifier, is widely utilized as a nutrient carrier and soil conditioner in agriculture. CMC reduces the surface tension of its solutions, improving the spreadability of the biopolymer formulations, in particular when Pluronic F-127 (PF-127) is added. Pluronics are triblock copolymers of polyoxyethylene (PEO; hydrophilic), and polyoxypropylene (PPO; hydrophobic), with the structure (PEO)a(PPO)b(PEO)a, which confers amphiphilic properties to the polymers. These formulations are widely used, due to their non-toxicity in the biomedical field, plus they have been tested in the agricultural context as a stabilizing agent in nanostructured systems for the controlled release of agrochemicals [37]. Many agricultural applications often require the presence of surfactants in order to improve the spreadability or dispersion of the formulations on hydrophobic plant surfaces (i.e., leaves, seeds or roots), a property that a CMC preparation could potentially provide. Furthermore, cellulose derivatives, such as CMC, exhibit the intrinsic advantages of cellulose: natural abundance, biodegradability and biocompatibility, combined with water solubility; all properties that integrate well with the concept of a green circular economy in agriculture.

This study aims to evaluate the effect of different biological treatments on the quantitative parameters associated with yield and the qualitative characteristics, as determined by analysis of metabolomic profiles, of mini plum tomato. The tested constituents included *T. harzianum* T22 and a biopolymer, consisting of carboxymethyl cellulose and a nonionic synthetic surfactant, that were applied singly or in combination to the plants. In addition, the effect of the bioactive *Trichoderma* secondary metabolite 6PP was also evaluated in combination with this biopolymer

This investigation will be useful in determining the outcome of combining the *Trichoderma* living fungus or a *Trichoderma* secondary metabolite with a biopolymer of vegetal origin in a biostimulant formulation that could have promising results for improving the yield components and nutritive characteristics of an important greenhouse horticultural crop, such as tomato.

## 2. Results

### 2.1. Yield, Yield Components, Shape Index and Juice Quality

**Figure 1:**
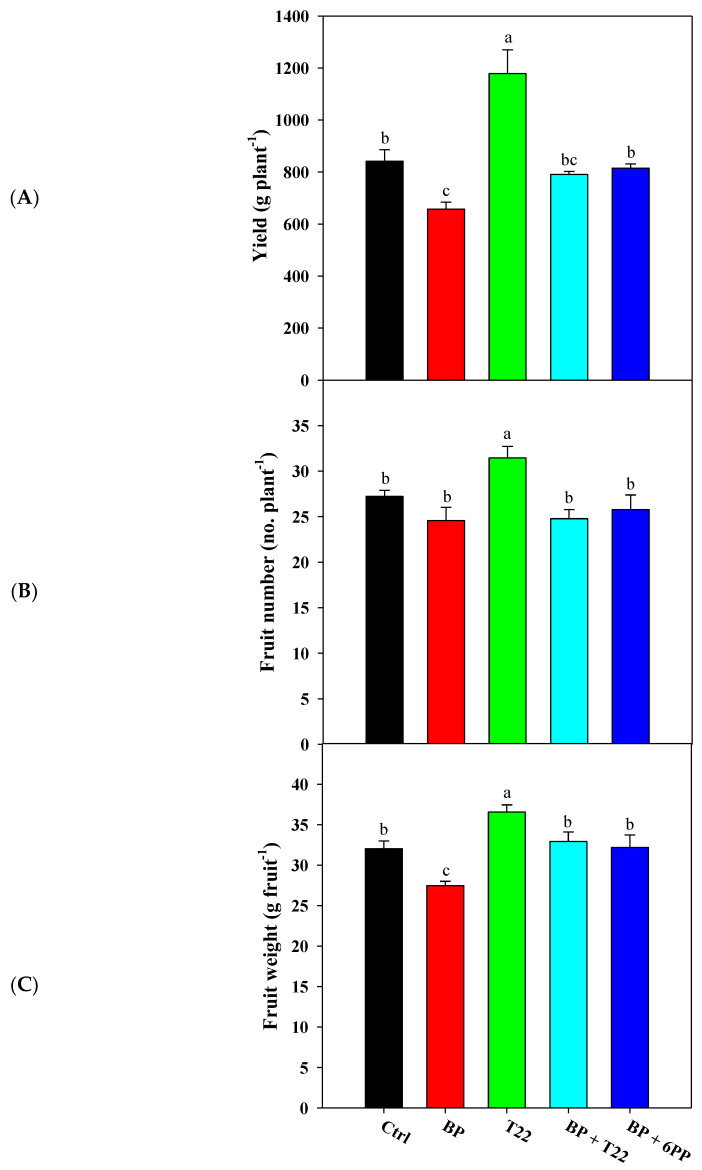
Effects of *Trichoderma harzianum* strain T22 (T22) or biopolymer (BP) applications alone, or in combinations of BP + T22 or BP + 6-pentyl-α-pyrone [6PP] (BP + 6PP), on fresh yield (**A**), fruit number per plant (**B**) and mean fruit weight (**C**) of greenhouse plum tomato. The control was not treated with biostimulants (Ctrl). Values are the means of three replicates ± SE. Different letters indicate significant differences according to Duncan’s test (*p* = 0.05).

The results indicated effects of *Trichoderma*, metabolite and biopolymer (BP) applications on crop productivity, whereby inoculations with *Trichoderma harzianum* strain T22 elicited a significant increase in fruit yield (by 40.1%) in comparison to the untreated control (Figure 1), and a greater increase of 79.3% over the BP treatment alone. However, the beneficial effects of the *Trichoderma*, fungus or metabolite on yield parameters were not apparent in any of the combined BP + T22 or BP + 6PP treatments inoculated to the tomato plants, which were not significantly different from the control (Figure 1). Interestingly, the higher production observed in greenhouse tomato plants inoculated with T22 was attributed to both an increase in the mean fruit weight, and to a lesser extent, to a higher number of fruit produced per plant (Figure 1).

No significant differences among treatments were recorded for the fruit dry matter content (avg. 7.9%), total soluble solids (TSS) (avg. 6.7 °Brix) and juice pH (avg. 4.4), whereas the shape index (SI) and juice electrical conductivity (EC) were significantly affected by the *Trichoderma*, metabolite or BP applications (Table 1). The SI and juice EC ranged from 0.77 to 0.81 and from 3.5 to 4.4. dS m^−1^, respectively, with the lowest values recorded in BP + 6 PP treatments (Table 1). Higher juice EC values are usually correlated with higher total soluble solids and organic acids, parameters determining the taste properties of tomato [38].

### 2.2. Carbohydrates and Bioactive Metabolites Content

The applications of T22 or BP alone or in combination, as a well as BP + 6PP treatments, did not cause a significant effect on starch (avg. 10.5 μmol g^−1^ dw), glucose (avg. 170.1 μmol g^−1^ dw), fructose (avg. 34.7 μmol g^−1^ dw), sucrose (avg. 12.6 μmol g^−1^ dw) or polyphenols (avg. 5.0 mg GAE g^−1^ dw) in the greenhouse tomato fruits (Table 2). However, only the content of lycopene, the major lipophilic antioxidant in tomatoes, was significantly increased (*p* < 0.001) upon applications with the fungus or the metabolite compared to the untreated control, as shown for T22 alone (+ 49%), BP + T22 (+40%) and BP + 6PP (+52%), with no significant differences noted between the two combined treatments (Table 2). Specifically, the lycopene content in the tomato fruits varied from 1.64 mg g^−1^ dw in the control to 2.45 and 2.50 mg g^−1^ dw in treatments with T22 alone and BP + 6PP, respectively.

### 2.3. Soluble Proteins and Free Amino Acids Contents

Soluble protein content in control treatments was 20.7 mg g^−1^ dw and this parameter was significantly increased by more than 20% with treatments of BP and BP + T22 (Table 3). In the evaluation of the mean total amino acids, it was observed that glutamate alone (avg. 187 μmol g^−1^ dw) accounted for 58.2% of the overall content, irrespective of the tomato treatments. Furthermore, glutamine (avg. 30.2 μmol g^−1^ dw), aspartate (avg. 26.0 μmol g^−1^ dw), asparagine (avg. 13.3 μmol g^−1^ dw), GABA (avg. 13.7 μmol g^−1^ dw) and arginine (avg. 11.3 μmol g^−1^ dw), even if detected in lower concentrations, all together represented 29.4% of the mean total amino acid content in all treatments (Table 3). Essential amino acids (the sum of arginine, histidine, isoleucine, leucine, lysine, methionine, phenylalanine, threonine, tryptophan and valine) accounted for 10% of total amino acids independently of treatment. In general, the single or combined applications of *Trichoderma*, metabolites or BP did not change the total amino acid content (avg. 321 μmol g^−1^ dw). However, it should be noted that all treatments resulted in a highly significant decrease (*p* < 0.001) in the glycine content in comparison to the control, from an initial value of 3.48 μmol g^−1^ dw in the control and a decrease to an average value of 0.19 μmol g^−1^ dw with the T22, BP + T22 and BP + 6PP applications (Table 3). In particular, it can be observed that the T22 treatment alone significantly increased (*p* < 0.05) the content of asparagine (+37%), GABA (+87%) and MEA (+102%) compared to the untreated control. On the other hand, BP alone was also noted to strongly increase the content of GABA (+105%) and MEA (+85%). No other significant changes were induced by the single applications of *T. harzianum* strain T22 or BP alone or in the combinations (BP + T22 or BP + 6PP).

### 2.4. Principal Component Analysis

**Figure 2:**
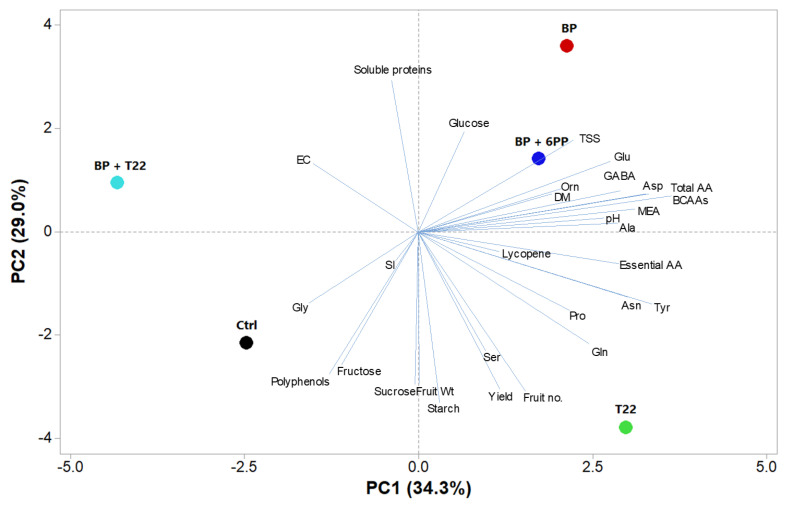
Principal component loading plot and scores of principal component analysis (PCA) of morphological parameters, starch, soluble carbohydrates, polyphenols, lycopene and soluble protein and amino acid profiles of greenhouse tomato as affected by different treatments: *Trichoderma harzianum* strain T22 (T22) or biopolymer (BP) alone or in combination (BP + T22 or BP + 6PP). The control was not treated with biostimulants (Ctrl).

To obtain an overview of crop productivity and the compositional profile of greenhouse tomato with the different *Trichoderma*, metabolite or BP applications, a principal component analysis (PCA) was performed on all measured parameters (Figure 2). The first three principal components (PCs) were related, with eigenvalues higher than 1, and explained 85.3% of the total variance, with PC1, PC2 and PC3 accounting for 34.3%, 29.0% and 22.0%, respectively (data not shown). PC1 was positively correlated to total amino acids, essential amino acids, particularly branched chain amino acids (BCAAs), tyrosine, aspartate, asparagine, MEA, GABA, alanine, glutamate, glutamine and juice pH. PC1 was also negatively correlated to glycine and juice EC. Moreover, PC2 was positively correlated to soluble proteins, TSS and glucose, while PC2 was negatively correlated to starch, fruit number, fresh yield, sucrose, fruit mean weight, polyphenols, fructose and serine (Figure 2). The treatments were well separated and distributed in the loading plot. The control was positioned in the middle of the lower left quadrant, strongly correlated to glycine content. The combination of BP + T22 was positioned on the negative side of PC1 in the upper left quadrant, highly correlated with EC (Figure 2). Treatments with BP + 6PP and BP were positioned on the positive side of PC1 in the right upper quadrant, correlated to soluble proteins, TSS, glutamate and GABA. Meanwhile, T22 treatment was in the lower right quadrant on the negative side of PC2 and was characterized by high yield and yield components and high starch, glutamine, proline tyrosine, asparagine and serine contents (Figure 2). The distribution of the treatments across the measured factors in PC2 was strongly attributed to the presence of the biopolymer.

## 3. Discussion

The use of *Trichoderma* spp., or other endophytic fungi, as a promising tool and sustainable approach in modern agriculture, has emerged in the last twenty years due to the multifaceted properties and positive effects observed on horticultural and agricultural crops [14,16,39]. Hence, the recent movement by researchers, farmers and biotechnological companies to evaluate new *Trichoderma*-based products developed to enhance and optimize bioformulations with beneficial fungi and/or their bioactive metabolites on crops by using novel delivery systems [9,12,15]. To this end, the present study tested and demonstrated the effectiveness of a biopolymer composed of vegetal carboxymethyl cellulose and synthetic Pluronic F-127 used alone, but more importantly in combination with *T. harzianum* T22, as well as with 6PP, an active *Trichoderma* metabolite known to exert multiple beneficial effects on plants, on the production of Pixel tomatoes in the greenhouse.

The first important confirmation was that inoculation of the Pixel tomato with *Trichoderma harzianum* T22 significantly improved the marketable yield, fruit mean weight and number of fruits per plant compared to untreated plants. This effect could be attributable to several concomitant beneficial effects elicited by this fungus, including crop protection from pathogens [9] and the growth promotion of root biomass and architecture, as well as increased nutrient uptake and transport (i.e., soil acidification, and Fe and Cu chelation) [40]. Compounds similar to plant hormones, such as indole-3-acetic acid (IAA), indole-3-acetaldehyde (IAAld) and indole-3-ethanol (IEt), are synthetized by *Trichoderma* spp. [41], which stimulate lateral root formation, thus increasing the root surface area, creating niches for fungal colonization, as well as indirectly improving the water and nutrient uptake capacity of the plant and the processes associated with photosynthesis [9,12,15,19,42]. Although the secondary metabolite 6PP was previously reported to exert an auxin-like activity and promote plant growth [33], it did not produce the significant effects on tomato marketable yield and fruit production as noted with the use of the living *Trichoderma* fungus in this study. The fungus is probably able to not only to enhance the physiological active uptake mechanisms of the plant, but to act directly on the solubilization of various nutrients in the soil rhizosphere, such as rock phosphate, iron, copper and divalent ions, or to produce siderophores, which render these elements available to the plant [14]. The positive biological activities on the plant were inhibited in presence of BP, since fruit growth and yield observed with *Trichoderma* alone were significantly higher, but when the fungus or the metabolite were combined with BP, the beneficial action on the greenhouse tomato was no longer obtained.

In contrast, the treatment with BP alone, or in combination with *T. harzianum* T22, determined a significant increase in protein content in the tomatoes, probably due to the effect on metabolic processes that increase NUE. In fact, carboxymethyl cellulose hydrogel is known to facilitate nutrient availability in the rhizosphere, enhancing not only nitrogen uptake, but in particular the absorption of important bivalent ions by the plant [43]. For example, magnesium (Mg^2+^) is the central atom in the tetrapyrrole ring of chlorophyll molecules and can act as an activator of more than 300 enzymes, thus playing a key role in various physiological and biochemical processes, including chlorophyll biosynthesis and photosynthesis [44]. In addition, manganese (Mn^2+^) is necessary for chlorophyll synthesis, but above all, it plays a key role in the oxygen evolving complex (OEC) of photosystem II, since four atoms of Mn^2+^ are required to split water [45]. Therefore, the uptake of these elements by the plant contributes to the metabolic processes that influence the quality of the fruits.

The treatment with *T. harzianum* T22 also demonstrated an improvement in nitrogen metabolism in plum tomato, as noted by the increase in the asparagine content, the most common amide used for the long-distance transport of nitrogen throughout the plant. This effect could be due to the ability of fungi, like *Trichoderma* spp., to promote the uptake of nitrogen, as observed in field grown corn [14], or to increase the expression of genes encoding for key enzymes involved in the assimilatory reduction pathway, such as nitrate reductase (NR) [46,47]. Furthermore, the increase in asparagine and the strong decrease in glycine, a photorespiratory amino acid, were not accompanied by a statistically significant decrease in the starch or soluble protein content in fruits that could imply that *T. harzianum* T22 was able to increase the photosynthetic efficiency while decreasing the photorespiratory activity of the treated plant. Alternatively, it is possible that the fungus maximized the re-assimilation of photorespiratory products through a rapid re-use of glycine and ammonium released during photorespiration, thus resulting in a de novo synthesis of amides [48].

In addition, both of the single treatments of *T. harzianum* T22 and BP revealed a significant increase in GABA, a non-protein amino acid that can act as a temporary deposit of nitrogen to reduce the accumulation of excess ammonium produced by photorespiration, thus allowing the stationary control of pH, since protons are consumed in the process [49]. The synthesis of GABA, catalyzed by the enzyme glutamate decarboxylase (GAD), releases CO_2_, which allows the Calvin cycle to function with closed or semi-closed stomata under drought stress conditions, thus reducing the pressure on the photosynthetic electron chain and decreasing ROS and photodamage in plant tissues [50]. The zwitterion form of GABA is able to act as an osmolyte without toxic effects in balancing the decrease in water potential during cellular dehydration and functioning as an antioxidant for the stabilization and protection of thylakoids and macromolecules [50]. Furthermore, it has been reported that GABA has many beneficial effects on human health, including hypotensive effects, the enhancement of immune functions under stress, the prevention of cancer and diabetes and control of blood cholesterol levels [51,52,53].

The increase in GABA did not determine a decrease in its precursor, glutamate, which remained stable except for a non-significant decrease with the treatment BP + T22. The stability of glutamate content is of primary importance since it can affect tomato flavor and fruit palatability, as it has been reported to elicit an intense umami taste [54].

*Trichoderma harzianum* T22 and BP alone, as well as the BP + 6PP treatment, also determined an increase in the monoethanolamine (MEA) content, an amino acid derivative important for the synthesis or regeneration of phospholipids, derived by the decarboxylation of the photorespiratory amino acid serine. Therefore, the application of these treatments allowed tomato plants to reuse the photorespiratory amino acids and ammonium to synthesize useful metabolites, thus reducing the pressure of photorespiration on plant metabolism in favor of optimizing the processes involved in photosynthesis.

The content of lycopene significantly increased with all the tested treatments that included *T. harzianum* T22 or the fungal secondary metabolite 6PP. It is well documented that lycopene is the main lipophilic antioxidant found in tomato fruits, and its increase is particularly important because it could assure a longer shelf-life of plum tomatoes. On the contrary, polyphenols that behave as free radical scavengers and take part in redox reactions due to their ability to transport protons and electrons and confer resistance to abiotic stresses and pathogens [54,55], were not affected by *Trichoderma*, metabolites or BP applications, as recently found by Caruso et al. [56]. This result could mean that there is a basal constitutive amount of polyphenols in plum tomatoes that is independent of influences by external stimuli. Lycopene present in tomato products also has important human health properties. It can be involved in protection against chronic disorders, such as cardiovascular disease or prostate, respiratory and digestive epithelial cancers [57]. Recent epidemiological data have proven that it exerts cardiovascular protection by decreasing High-Density Lipoprotein (HDL) -associated inflammation and modulating HDL functionality [58]. Moreover, lycopene can scavenge ROS, in particular hydroxyl radicals, and stimulate antioxidant enzymes, such as superoxide dismutase, glutathione peroxidase and glutathione reductase, thus preventing oxidative stress-mediated carcinogenesis [59,60,61]. 

The development of biologically based products for agriculture is complex due to the mix of components in the formulation, particularly if they include living microorganisms. In nature, the interactions among plants, microbes and the substances in the environment are multiple, various and influence the agroecosystem of crop production in diverse and often unpredictable ways.

## 4. Materials and Methods

### 4.1. Biostimulants, Plant Material and Greenhouse Experimental Design

Four biostimulant treatments were used: (i) *Trichoderma harzianum* strain T22 (T22; the commercial formulation of Trianum-P, Koppert Biological Systems; used at the label recommendation of a final concentration 10^7^ spore mL^−1^), (ii) biopolymer (BP; diluted in water), (iii) biopolymer + T22 (10^7^ spore mL^−1^) and (iv) biopolymer + 6-pentyl-α-pyrone (6PP; concentration 10^−6^ M). A metabolite derived from *Trichoderma* sp., 6PP, was purchased from Sigma Aldrich (St. Louis, MO, USA). The biopolymer formulation was a combination of: carboxymethyl cellulose (CMC; average molecular weight (Mw) of 250 kDa) and Pluronic F127 (PF-127; (PPO)100(PEO)65(PPO)100, Mw = 12500 g mol^−1^; Sigma-Aldrich, Milan, Italy). A stock biopolymer solution was prepared by solubilizing CMC (1% *v/v*) and PF-127 (1% *v/v*) in water with mechanical stirring for 4 h, in order to achieve the complete dissolution of the polymers. The stock solution was then diluted with water (1:1) before adding T22 spores or 6PP, prior to plant application in the field.

Mini plum tomato (*Solanum lycopersicum* L.) seedlings variety Pixel F1 (indeterminate growth; ISI Sementi SpA, Fidenza, Italy) were transplanted to a polyethylene tunnel greenhouse located at the University of Naples Federico II, Portici (NA), south Italy (40°49′ N, 14°15′ E; 72 m a.s.l.) on 4 April 2019. Prior to transplant, 1.4 kg m^−2^ of manure (32-7-2) was added to the soil and mixed. At the time of transplant, the seedling roots were dipped in solutions containing the four separated liquid inocula and the controls were treated with water. The tomato plants were transplanted in single rows at 1 m distance between rows, while within the rows, tomato seedlings were at a 30 cm distance, accounting for a density of 3.3 plants m^−2^.

The four treatments and the control were arranged in a randomized complete block design with three replicates, which consisted of fifteen plants each. The biostimulant treatments were repeated on 16 May, 7 June and 4 July by manually watering each plant with the inoculum at volumes of 25, 100 and 250 mL, respectively.

The plants were irrigated using a drip irrigation system, and during the growing cycle, 2.5 kg of calcium nitrate (Calcinit, YaraLiva, Milano, Italy) was applied three times via fertigation in two-week intervals, starting on 31 May. Foliar treatments consisted of a mix of calcium and magnesium (Calimag, Meristem, Moncada, Spain) applied between mid-June and beginning of July, while *Bacillus thuringiensis* vr. *kurstaki* (BAC MK, Comercial Química Massó, S.A., Barcelona, Spain) was applied four times, in 10-day cycles, in intervals starting in the beginning of June.

### 4.2. Yield Components, Sampling and Fruit Quality Assessments

Harvesting was performed once weekly starting on 12 July to determine the yield and yield components. Fruits were collected from the 3rd cluster for determining fruit shape index and performing the qualitative analysis. Throughout the harvest period, the marketable yield, the number of fruits per plant and the mean fruit weight were measured on forty-five plants from each treatment. Deformed or misshaped fruits were discarded because of unmarketable quality. A subsample of fresh fruits was used for the determination of shape index, total soluble solid content, juice pH and electrical conductivity (EC), while the remaining subsample was shock-frozen in liquid nitrogen, lyophilized and stored at −80 °C until further chemical analyses of: starch, soluble carbohydrates, polyphenols, lycopene, soluble proteins and free amino acids.

The shape index was determined as the ratio of fruit equatorial and meridian diameters. Mini plum tomato fruits were homogenized in a Waring^®^ blender (2 L capacity; Model HGB140, CA, USA) for 1 min, the liquid was filtered through a double cheesecloth to remove the pulp, then the juice was subjected to measurements of total soluble solid (TSS) content with a digital Atago N1 refractometer (Atago Co. Ltd., Tokyo, Japan), juice pH was measured by a HI-9023 pH meter (Hanna Instruments, Padova, Italy) and juice EC was evaluated by a HI-99301 EC meter (Hanna Instruments, Padova, Italy). Fruit dry matter content was assessed as a percentage of fresh mass after desiccation at 75 °C for about 3 days, until constant weight was achieved.

### 4.3. Starch and Soluble Carbohydrate Analysis

Soluble sugars (µmol g^−1^ dw) were estimated in the supernatant of ethanolic extracts of lyophilized tomato samples by a coupled enzyme assay based on NAD-linked enzymatic reactions and the determination of NADH at 340 nm recorded by a Synergy HT spectrophotometer (BioTEK Instruments, Bad Friedrichshall, Germany) [54]. This assay was also used to quantify starch in the pellets of the same ethanolic extracts after hydrolysis to glucose, according to Carillo et al. [54].

### 4.4. Polyphenol and Lycopene Analysis

The total polyphenol content was determined by using the Singleton [62] Folin–Ciocalteu method with modifications, as described in Carillo et al. [63]. The polyphenol concentration was expressed as gallic acid equivalents (GAE) (mg GAE g^−1^ dw).

Lycopene concentration (mg g^−1^ dw) was evaluated according to Sadler et al. [64] from lyophilized tomato samples (15 mg) using the modifications described in Carillo et al. [54].

### 4.5. Soluble Proteins, Free and Total Amino Acid Analysis

Soluble proteins (mg g^−1^ dw) were extracted from 10 mg of lyophilized tomato plant material with 1 mL of 200 mM Tris-HCl 200 mM pH 7.5 containing 500 mM MgCl_2_ at 4 °C for 24 h, and assayed by the Bio-Rad Protein Assay, based on the Bradford method [63].

Primary amino acids and proline (µmol g^−1^ dw) were extracted from 10 mg of lyophilized fruits in 1 mL ethanol:water (40:60 *v/v*) overnight at 4 °C, and determined by HPLC after pre-column derivatization with o-phthaldialdehyde (OPA), according to Carillo et al. [54]. Proline was estimated in the same ethanol extract through an acid ninhydrin-based method modified by Woodrow et al. [48].

### 4.6. Statistics, Principal Component Analysis and Percentage Increase

A one-way analysis of variance (ANOVA) of all data was performed using the software package SPSS 13 for Windows 2001. Duncan’s multiple-range test was used to analyze separated means with a cut-off for statistical significance at *p* < 0.05. A principal component analysis was employed to investigate how the dominant quanti-qualitative parameters set clustered according to the treatments by using Minitab^®^ 18 statistical software (Minitab LLC, State College, PA, USA) [27,65]. The percentage increases were calculated as the difference between the treated sample values minus untreated sample values (increase), divided by the untreated sample values and multiplied by 100.

## 5. Conclusions

Company R&D programs often have a single objective for the final commercial product, and that is to satisfy the demands for increased crop yield or improved productivity from the producers. Although the interests of the consumer may be well satisfied by the wide assortment and availability of goods in the supermarket, their interests may also be aimed at the evaluation of the qualitative and health properties of the purchased food product. Our greenhouse experiment on plum tomato confirmed the ability of *T. harzianum* T22 to improve crop productivity (+40%) and the quality of the produce in terms of lycopene, asparagine and GABA compared to the untreated control. However, the other treatments were also able to increase the content of GABA or lycopene, enhancing the premium quality of plum tomatoes. Therefore, this investigation into the development of new bioformulations not only confirmed that *T. harzianum* T22 or BP alone, or in combination with T22 + BP or 6PP + BP, not only represent a promising strategy for improving the yield and quality of horticultural crops such tomato, but also demonstrate that in the absence of apparent increased productivity in the plant, some treatments are still able to produce beneficial nutritional effects in the fruit. The synthesis of bioformulations, based on findings from research such as the present study, aid in the establishment of sustainable cropping systems, as well as in the development of biofunctional foods, thus supporting the direction of agricultural advancement in the future.

## Figures and Tables

**Table 1:** Effects of treatments with *Trichoderma harzianum* strain T22 (T22) or biopolymer (BP) alone, or in combinations of BP + T22 or BP + 6-pentyl-α-pyrone (6PP) (BP + 6PP) on shape index, fruit dry matter content, total soluble solids (TSS) content, and juice pH and electrical conductivity (EC) of greenhouse Pixel tomato fruits. The control was not treated with biostimulants (Ctrl).

Treatment	Shape Index	Dry Matter Content	TSS	pH	EC
		(%)	(°Brix)		(mS cm^−1^)
Ctrl	0.81 a	8.03	6.73	4.40	3.83 ab
BP	0.81 a	8.38	7.03	4.42	4.37 a
T22	0.79 ab	8.00	6.63	4.39	3.87 ab
BP + T22	0.78 ab	7.67	6.47	4.33	4.48 a
BP + 6PP	0.77 b	7.79	6.80	4.40	3.48 b
Significance	*	NS	NS	NS	*

NS, non-significant or *, significant at *p* ≤ 0.05. Different letters within each column indicate significant differences according to Duncan’s multiple-range test (*p* = 0.05).

**Table 2:** Effects of applications of *Trichoderma harzianum* strain T22 (T22) or biopolymer (BP) alone or in combinations (BP + T22 or BP + 6PP) on the content of starch, glucose, fructose, sucrose, polyphenol and lycopene in greenhouse Pixel tomato fruits. The control was not treated with biostimulants (Ctrl).

Treatment	Starch	Glucose	Fructose	Sucrose	Polyphenols	Lycopene
	(µmol g^−1^ dw)	(µmol g^−1^ dw)	(µmol g^−1^ dw)	(µmol g^−1^ dw)	(mg GAE g^−1^ dw)	(mg g^−1^ dw)
Ctrl	12.57	166.2	42.40	13.96	5.10	1.64 b
BP	8.70	175.2	31.21	12.04	4.64	1.86 b
T22	13.26	170.2	36.23	13.58	5.39	2.45 a
BP + T22	9.19	171.9	32.42	11.90	5.25	2.29 a
BP + 6PP	8.96	167.3	31.27	11.69	4.67	2.50 a
Significance	NS	NS	NS	NS	NS	***

NS, non-significant or ***, significant at *p* ≤ 0.001. Different letters within each column indicate significant differences according to Duncan’s multiple-range test (*p* = 0.05).

**Table 3:** Effects of *Trichoderma harzianum* strain T22 (T22) or biopolymer (BP) applications alone or in combinations (BP + T22 or BP + 6PP) on soluble proteins, free amino acid profile and total amino acid content of greenhouse tomato fruits. The control was not treated with biostimulants (Ctrl).

Chemical Compound	Treatment					Significance
	Ctrl	BP	T22	BP + T22	BP + 6PP	
Soluble proteins (mg g^−1^ dw)	20.66 c	24.47 ab	21.47 bc	24.75 a	23.46 abc	*
Alanine (µmol g^−1^ dw)	6.23	6.49	6.64	5.57	7.44	NS
Arginine (µmol g^−1^ dw)	10.34	8.52	12.75	10.77	14.09	NS
Asparagine (µmol g^−1^ dw)	11.66 b	13.58 b	16.02 a	12.01 b	13.05 b	*
Aspartate (µmol g^−1^ dw)	25.15	28.12	26.46	21.71	28.42	NS
GABA (µmol g^−1^ dw)	9.07 c	18.62 a	17.00 ab	11.39 bc	12.53 bc	*
Glutamine (µmol g^−1^ dw)	28.49	29.82	37.35	27.12	28.27	NS
Glutamate (µmol g^−1^ dw)	179.9 ab	231.3 a	186.6 ab	147.9 b	189.1 ab	*
Glycine (µmol g^−1^ dw)	3.48 a	0.18 b	0.15 b	0.15 b	0.27 b	***
Histidine (µmol g^−1^ dw)	4.72	4.90	5.01	4.48	5.03	NS
Isoleucine (µmol g^−1^ dw)	1.58	1.93	1.90	1.55	1.90	NS
Leucine (µmol g^−1^ dw)	2.29	2.84	2.71	2.17	2.75	NS
Lysine (µmol g^−1^ dw)	1.62	1.59	1.69	1.33	1.62	ns
MEA (µmol g^−1^ dw)	0.46 b	0.86 a	0.94 a	0.65 ab	0.81 a	*
Methionine (µmol g^−1^ dw)	0.40	0.48	0.45	0.37	0.42	NS
Ornithine (µmol g^−1^ dw)	1.18	1.23	1.19	1.07	1.52	NS
Phenylalanine (µmol g^−1^ dw)	5.61	6.92	6.46	5.21	7.18	NS
Proline (µmol g^−1^ dw)	4.57	4.91	5.77	4.99	5.03	NS
Serine (µmol g^−1^ dw)	2.90	2.32	2.67	1.86	2.18	NS
Threonine (µmol g^−1^ dw)	1.86	1.54	2.04	1.23	1.80	NS
Tryptophan (µmol g^−1^ dw)	0.73	1.11	0.75	0.75	0.92	NS
Tyrosine (µmol g^−1^ dw)	1.82	1.99	2.30	1.69	2.01	NS
Valine (µmol g^−1^ dw)	0.88	1.13	1.11	0.83	0.99	NS
Total amino acids (µmol g^−1^ dw)	304.9	370.4	337.9	264.8	327.3	NS

NS, non-significant or *, ***, significant at *p* ≤ 0.05, 0.001, respectively. Different letters within each column indicate significant differences according to Duncan’s multiple-range test (*p* = 0.05).
